# A *Streptococcus suis* Strain Δ*cps/ssna*-m*sly* (P353L)-SC19 Provides Cross-Protection against Serotypes 2 and 9 Strain Challenges in a Mouse Model

**DOI:** 10.3390/vaccines12030283

**Published:** 2024-03-08

**Authors:** Xi Lu, Lei Xu, Lan Lin, Liting Zhou, Bingqian Dai, Shuyue Cui, Anding Zhang

**Affiliations:** 1 State Key Laboratory of Agricultural Microbiology, Hubei Hongshan Laboratory, College of Veterinary Medicine, Huazhong Agricultural University, Wuhan 430070, China; lucylook5@webmail.hzau.edu.cn (X.L.); leixu@webmail.hzau.edu.cn (L.X.); lin_lan@hust.edu.cn (L.L.); 2019302010180@webmail.hzau.edu.cn (L.Z.); daibingqian@webmail.hzau.edu.cn (B.D.); cuisy@webmail.hzau.edu.cn (S.C.); 2 Key Laboratory of Preventive Veterinary Medicine in Hubei Province, The Cooperative Innovation Center for Sustainable Pig Production, Wuhan 430070, China; 3 Wuhan Institute of Virology, Chinese Academy of Sciences, Wuhan 430070, China; 4 Center for Biosafety Mega-Science, Chinese Academy of Sciences, Wuhan 430070, China; 5 Key Laboratory of Development of Veterinary Diagnostic Products, Ministry of Agriculture of the People’s Republic of China, Wuhan 430070, China; 6 International Research Center for Animal Disease, Ministry of Science and Technology of the People’s Republic of China, Wuhan 430070, China

**Keywords:** cross-protection, CPS-deficient, attenuated vaccine, *Streptococcus suis*

## Abstract

*Streptococcus suis* is an important zoonotic pathogen that mainly causes meningitis, septicemia, and arthritis. Due to the limited cross-protection between numerous serotypes, the existing inactive vaccines in clinical use fail to offer sufficient protection. In this study, a gene deletion-attenuated strain Δ*cps/ssna*-m*sly* (P353L)-SC-19 was constructed by deleting *cps* and *ssna* genes from the epidemic strain SC-19 with a mutation of SLY (P353L). The safety of Δ*cps/ssna*-m*sly* (P353L)-SC-19 was confirmed in both in vitro and in vivo experiments. We further demonstrated that immunization with Δ*cps/ssna*-m*sly* (P353L)-SC-19 induced significant cellular immunity and humoral immunity in mice and protected against infections caused by type 2 strain SC-19 (100% protection) and type 9 strain S29 (50% protection), while also preventing meningitis induced by S29. This study highlights the potential of using CPS-deficient strains to achieve cross-protection against different *Streptococcus suis* serotypes and develop a promising universal live vaccine.

## 1. Introduction

*Streptococcus suis* is an important zoonotic pathogen that mainly causes meningitis, septicemia, endocarditis, endophthalmitis, and arthritis [1,2,3]. Its impact is not limited to causing substantial economic losses in intensive pig production but also extends to human infections and fatalities, posing a serious threat to public health [4,5]. Among the 29 confirmed serotypes classified according to the variations in capsular antigens [6], *S. suis* serotype 2 (SS2) is considered the most prevalent serotype, with the highest pathogenicity in both humans and pigs [2,7]. It was responsible for the outbreak of human *S. suis* epidemics in Jiangsu, China, in 1998 and Sichuan, China, in 2005 [8]. Notably, these two outbreaks were characterized by the emergence of streptococcal toxic shock-like syndrome (STSLS), featuring abrupt onset, a short course of the disease, and an extremely high mortality [9]. In addition, *S. suis* serotype 9 (SS9) is now regarded as the predominant serotype in certain European countries and the second prevalent serotype globally [10], raising increasing concerns.

Vaccination is recognized as an important method to control *S. suis* in pigs, which can effectively reduce the use of antibiotics [11]. However, the presence of numerous serotypes and sequence types of *S. suis*, along with the lack of cross-protection among them, has created an urgent demand for the development of a universal vaccine, posing a significant challenge for researchers. Presently, most bacterins only offer protection against infections caused by the same serotype of *S. suis* [12]. Additionally, subunit vaccines usually require a combination with effective adjuvants [12], while the attenuated vaccines, identified through traditional methods, have the potential risk of reversion to virulence. Conversely, live-attenuated vaccines, constructed by deleting key virulence genes, are safe and can be distinguished from wild types, and thus is a promising method.

Selection for virulence genes among hundreds of them is a crucial step. As the outer shell of *S. suis*, capsular polysaccharides (CPS) can mask cell wall antigens and resist phagocytosis to evade the host’s immune response, thus representing the most critical virulence factor [13]. The virulence of the CPS-deficient strain decreased significantly both in vivo and in vitro [14], potentially exposing the common bacterial surface antigen shared by various serotypes. Therefore, the CPS-deleted *S. suis* may confer the ability to cross-protect the infection of other serotypes. In addition, suilysin (SLY) is also an important virulence factor capable of inducing cellular toxicity, disrupting complement-mediated phagocytosis and killing, increasing blood–brain barrier (BBB) permeability, eliciting host inflammatory responses, and promoting bacterial infection [15,16]. Moreover, it is also an important protective antigen that can stimulate a strong immune response in the host [17]. Previous studies conducted in our laboratory have demonstrated that a mutant strain containing P353L substitution in SLY (m*sly*(P353L)-SC19) lost its hemolytic activity and exhibited a significant reduction in pathogenicity in mice [18]. Consequently, by mutating the enzyme-active site of SLY, the virulence of the strain can be reduced while preserving the immunogenicity of SLY. In addition, *S. suis* secretory nuclease A (SsnA) is a virulence-related factor that is expressed throughout all growth phases of *S. suis* and is present in most clinical isolates and serotypes. It has been demonstrated to facilitate evasion from neutrophil extracellular traps (NETs) [19] and represents an appealing target for attenuated live vaccines [20].

Consequently, in this study, we knocked out the CPS based on the m*sly*(P353L)-SC19 strain to expose potential universal surface antigen, and knocked out the virulence-related factor SsnA to serve as a vaccine marker, thereby obtaining the vaccine candidate strain named Δ*cps/ssna*-m*sly* (P353L)-SC19, abbreviated to css-SC19. We assessed the safety of the vaccine candidate strain both in vivo and in vitro and evaluated its efficacy in providing protection against SS2 and its potential for cross-protection against SS9 infection, thereby establishing a theoretical foundation for the development of an attenuated vaccine against *S. suis.*

## 2. Materials and Methods

### 2.1. Bacterial Strains, Plasmids, and Growth Conditions

The *S. suis* epidemic strain SC-19 belongs to serotype 2, displaying high pathogenicity in humans, mice, and pigs. The *S. suis* serotype 9 strain 29 was isolated from a pig with meningitis in China*. S. suis* strains were cultured in Tryptic Soy Broth or Tryptic Soy Agar (Difco Laboratories) supplemented with 10% newborn bovine serum at 37 °C. *Escherichia coli* DH5α and Mc1061 were cultured in Luria–Bertani medium at 37 °C.

### 2.2. Ethics Statement

The experimental infectious studies were conducted strictly by following the Guide for the Care and Use of Laboratory Animals Monitoring Committee of Hubei Province, China. Additionally, the protocol received approval from the Scientific Ethics Committee of Huazhong Agricultural University (Permit Number: HZAUMO-2019-049). Every effort was made to minimize animal suffering.

### 2.3. Construction of Δcps/ssna-msly (P353L)-SC19

The construction of css-SC19 was based on the SLY (P353L) mutant strain constructed by our laboratory [18]; *ssna* and *cpsEF* were deleted sequentially using the strategy of allelic replacement mutagenesis described before [21,22]. Briefly, the sequence of *ssna* was cloned into the temperature-sensitive *S. suis-E.coli* shuttle vector, pSET4s, and the plasmid psET4s-Δ*ssna* was introduced into Δ*cpsEF*-SC-19 by electroporation. The Δ*ssna* mutant strain was selected by spectinomycin. Subsequently, the sequence flanking *cpsEF* was cloned into pSET4s and was electroporated into Δ*ssna*-m*sly* (P353L)-SC-19 to generate Δ*cps/ssna*-m*sly* (P353L)-SC-19, thus the mutant Δ*cps/ssna*-m*sly* (P353L)-SC-19 strain was obtained. Supplementary Table S1 lists the primers used to construct the gene-deleting plasmids and to identify the corresponding mutant strains.

### 2.4. In Vitro Virulence Test

To generate the death model of human lung carcinoma cells (A549), we cultured them in F-12K medium supplemented with 10% FBS, and incubated at 37 °C with 5% CO_2_. A total of 8 h after treatment with Δ*cps/ssna*-m*sly* (P353L)-SC-19 or SC-19 (multipilicity of infection (MOI) = 20), A549 cells were harvested and stained by PI/ Annexin V-FITC, followed by flow cytometry analysis (BD Biosciences, San Jose, CA, USA).

### 2.5. Immunization and Experimental Infection of Mice

We divided 100 specific-pathogen-free female C57 mice, aged 4 to 6 weeks, into two groups. The first group received intraperitoneal immunization with 5 × 10^8^ CFU of the Δ*cps/ssna*-m*sly* (P353L)-SC-19 strain suspended in 500 μL of PBS, while the second group received intraperitoneal injections of 500 μL of PBS. Each mouse was immunized twice at 2 weeks interval.

Experimental infections were conducted as previously described [18]. Briefly, following the booster immunization, 10 mice from each group were intraperitoneally infected with SS2 strain SC-19 (4 × 10^8^ CFU) or SS9 strain S29 (5 × 10^6^ CFU). Subsequently, the mice were monitored daily for two weeks post-infection. Experimental infections were also conducted to assess the cytokine response, blood chemistry, bacteria burden, and H&E staining during *S. suis* infection. The mice received an intraperitoneal infection with 4 × 10^8^ CFU of SC-19 or 5 × 10^6^ CFU S29 and were euthanized 6 h or 24 h post-infection. The blood was collected for bacteria load detection, and the lung, liver, and brain were collected for H&E staining and bacterial load detection. For biochemistry analysis and inflammatory cytokines evaluation, the blood was prepared into plasma and then by using a VITALAB SE Chemistry Analyzer or U-Plex^TM^ Chemiluminescent ELISA (MSD, Rahway, NJ, USA).

### 2.6. Cytokine Detection and Lymphocyte Proliferation In Vitro

On day 28 of the first immunization, 3 mice per group were sacrificed, and the spleens were removed under aseptic conditions for lymphocyte proliferation and cytokine detection, as previously described [23]. In brief, the spleens were mashed and filtered through a 40 μm cell strainer, and RBCs were removed with ACK lysing buffer. Then, the splenocytes were resuspended in RPMI 1640 plus 20% of Fetal Calf Serum to the final concentration of 8 × 10^6^ cells/mL, and 100 μL cells were seeded in 96-well plates and incubated at 37 °C in a 5% CO_2_ incubator. The cells were stimulated by 4 × 10^7^ CFU inactivated SS2 strain, SC-19, or SS9 strain S29 (MOI = 50) for 72 h; concanavalin A (ConA) and 1640 served as the positive and negative controls, respectively. The cell supernatant was collected and assessed for IFN-γ content using an ELISA kit (Invitrogen, Waltham, MA, USA).

### 2.7. Opsonic Killing Assay

Mouse peritoneal macrophages were isolated from nonimmune SPF C57 mice following the method in a previous study [24]. Further, 1 × 10^3^ CFU bacteria were pre-incubated in 50 μL of immune or nonimmune mouse sera for 30 min at 37 °C. The mixtures were then added into 100 μL (1 × 10^5^) cell murine peritoneal macrophages and incubated for 1 h at 37 °C. The survival percentages of the *S. suis* strains incubated with nonimmune sera were considered 100%.

### 2.8. Quantification of Antibody Responses

Serum samples were collected on day 28 after immunization. Polyvinyl chloride 96-well plates were coated with 10^6^ CFU of SC-19 or S29, respectively, at 4 °C overnight, followed by blocking with 5% BSA for 1 h at 37 °C. Sera were then diluted in 5% BSA and incubated for 1 h at 37 °C. Subsequently, the plates were washed with PBS with Tween 20 and incubated with peroxidase-conjugated goat anti-mouse immunoglobulin IgG, IgG1, and IgG2b (Southern Biotech, Birmingham, AL, USA) for 45 min at 37 °C. After that, plates were coated with 3,3′,5,5′-Tetramethylbenzidine substrate for 20 min. The reaction was halted using 1 M H_2_SO_4_, and the absorbance was measured at 450 nm.

### 2.9. Evaluation of Blood–Brain Barrier Integrity

The permeability of the blood–brain barrier was determined as previously described [25], with some modifications. Briefly, on day 28 after the first immunization, 6 mice from the immunized group and control group were intraperitoneally infected with SS9 strain S29 (5 × 10^6^ CFU per mouse). At 60 h post-infection, 3 mice of each group were intravenously injected with 200 μL of 1% (*w*/*v*) Evans blue dye (Sigma, E2129-10G, Livonia, MI, USA) and perfused transcardially with PBS 15 min later under anesthesia. Then, the whole brain was removed, weighed, and homogenized, and the Evans blue dye was extracted using 50% (*w*/*v*) trichloroacetic acid. The supernatant was quantified using absorbance spectroscopy at 610 nm. Additionally, three mice from each group were euthanized 60 h after infection, and their brains were removed for H&E staining to observe the pathological alterations.

### 2.10. Statistical Analysis

All statistical analyses were conducted using GraphPad Prism version 6.0. The data were presented as mean ± standard deviation. An unpaired two-tailed Student’s *t*-test was employed to assess the statistical significance between the two groups. For comparisons among more than two groups, either one- or two-way analysis of variance with Tukey’s multiple-comparison test was utilized. Additionally, the log-rank test was applied to evaluate the statistical significance of survival curves.

## 3. Results

### 3.1. Construction of Δcps/ssna-msly (P353L)-SC19 and Its Biological Properties

**Figure 1:**
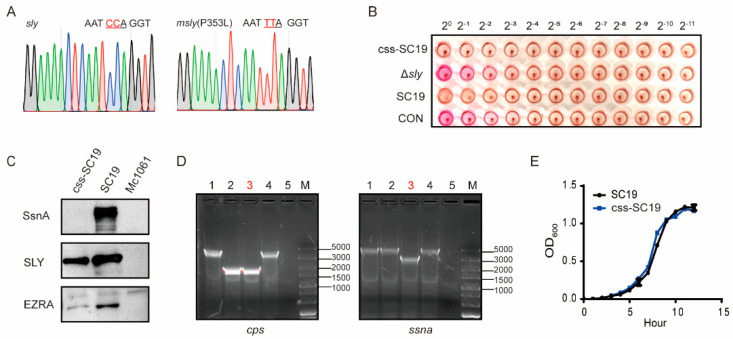
The basic characteristics of vaccine candidate strain css-SC19. (**A**) The sequencing of the *sly* gene from SC19 or css-SC19. (**B**) PCR confirmation of *cpsEF* and *ssna* gene deletion. 1: m*sly* (P353L)-SC19; 2: Δ*cps*-m*sly* (P353L)-SC19; 3: css-SC19; 4: SC19; 5: negative control; M: DL5000 Marker. (**C**) The content of SsnA and SLY was detected by Western blot, while EZRA served as a loading control and Mc1061 served as a negative control. (**D**) For the hemolytic activity of vaccine strain and Δ*sly* stain, a 50 μL culture supernatant of bacteria was incubated with chicken erythrocytes for 1 h after a series of 2-fold dilutions. (**E**) For the growth curve, the vaccine strain and WT strain were cultured at 37 °C, and the optical density at 600 nm was measured hourly.

To obtain an attenuated live vaccine strain, three genes (*cpsEF*, *ssna*, *sly*) that play crucial roles in the pathogenesis of *S. suis* were either deleted or mutated in the epidemic strain SC19 [18,21]. The mutant strain was designated *Δcps/ssna*-m*sly* (P353L), abbreviated as css-SC19, which still expressed the SLY but lacked hemolytic activity (Figure 1A–C). Furthermore, the deletions of *cpsEF* and *ssna* genes were confirmed through polymerase chain reaction (Figure 1D). The growth curve of the css-SC19 strain was similar to SC-19 (Figure 1E), indicating that the gene deletions of *cpsEF* and *ssna*, as well as the mutation of SLY (P353L), did not noticeably impact the basic biological characteristics.

### 3.2. Δcps/ssna-msly (P353L)-SC19 Exhibited Avirulent Features Both In Vitro and In Vivo

**Figure 2:**
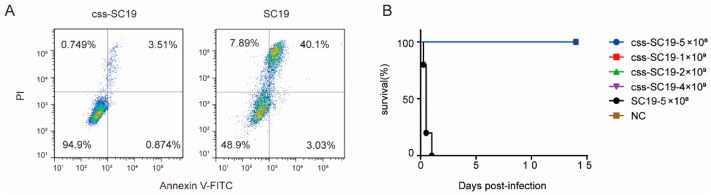
In vitro and in vivo virulence tests of css-SC19. (**A**) Cell death caused by css-SC19 or SC19. The incubation of css-SC19 or SC19 with A549 cells (MOI = 20) for 8 h at 37 °C and then stained by PI/Annexin V-FITC to determine cell death. (**B**) Survival of mice infected with SC19 or different doses of css-SC19. C57 mice received intraperitoneal infection with SC19 or different doses of the css-SC19 and were monitored for 14 days.

To evaluate the safety of css-SC19, A549 cells were treated with css-SC19 or SC19, respectively. The A549 cells treated with SC19 exhibited significant necrosis features, whereas those treated with css-SC19 showed no obvious necrosis features (Figure 2A).

To further evaluate the virulence of css-SC19, C57 mice were employed for the in vivo pathogenicity assessment. All mice infected with SC-19 succumbed within 24 h, while those infected with css-SC19 showed no clinical signs, and the survival rate was 100% (Figure 2B). Moreover, despite receiving a dosage eight times higher than a lethal dose of SC-19, all mice survived css-SC19 infection (Figure 2B).

These results indicate that css-SC19 exhibited avirulent characteristics both in vitro and in vivo, suggesting its potential as a live vaccine candidate.

### 3.3. Δcps/ssna-msly (P353L)-SC19 Could Induce Significant Humoral and Cellular Immune Responses

**Figure 3:**
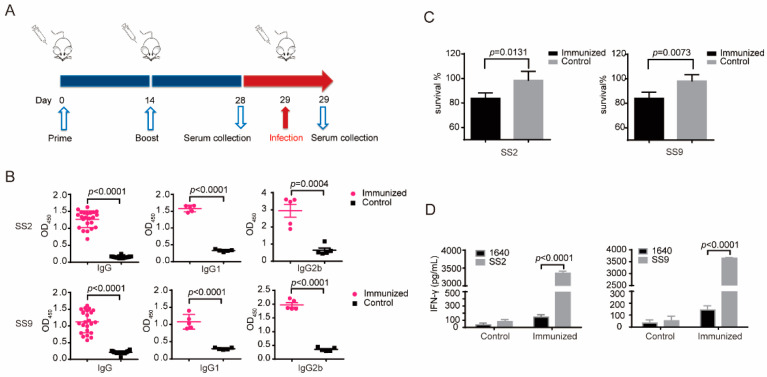
Immune responses induced by css-SC19 in mice. (**A**) The immunization and serum collection schedule. Mice were intraperitoneally immunized twice with css-SC19 (5 × 10^8^ CFU suspended in 500 μL PBS) at 2-week internal, while the placebo group was intraperitoneally injected with 500 μL PBS. Serum was collected for antibody detection on day 28. (**B**) Cytokine responses of spleen cells from immunized mice. Three mice from each group were sacrificed on day 28; the splenocytes were isolated under aseptic conditions and stimulated by inactivated SS2 and SS9 (MOI = 50), and the cell supernatant was collected for IFN-γ detection using an ELISA kit (Invitrogen). (**C**) Specific anti-SS2 and SS9 IgG, IgG1, and IgG 2b antibody levels. (**D**) The Opsonic killing of SS2 strain SC19 and SS9 strain S29.

Having confirmed the avirulent characteristics of css-SC19 both in vitro and in vivo, we conducted an assessment of the additional immune responses in mice that were immunized to css-SC19 (Figure 3A). The immunized mice exhibited significant levels of SC-19-specific and S29-specific IgG, IgG1, and IgG2b, with IgG2b being the predominant subclasses (Figure 3B), signifying a Th1-type bias. Given the importance of antibodies’ opsonophagocytosis-killing activity in resisting *S. suis* infection [26,27,28], we conducted an opsonophagocytosis-killing assay. The results revealed a 14.9356 ± 4.4235% and 14.3059 ± 5.0118% reduction of SC19 and S29 viability in the presence of immune sera of css-SC19 compared to the control sera (Figure 3C). Similarly, after stimulating spleen cells with a specific antigen in vitro, the cellular immune response can be assessed according to the variance in IFN-γ expression. Therefore, we evaluated the IFN-γ level of spleen cells from immunized or control mice following stimulation with SC19 or S29. The immunized group showed a substantial increase in IFN-γ levels after stimulation with SC-19 or S29 (Figure 3D), indicating that the immunization of css-SC19 could induce the cellular immune response against both SC19 and S29.

### 3.4. Δcps/ssna-msly (P353L)-SC19 Confers Protection against Type 2 S. suis

**Figure 4:**
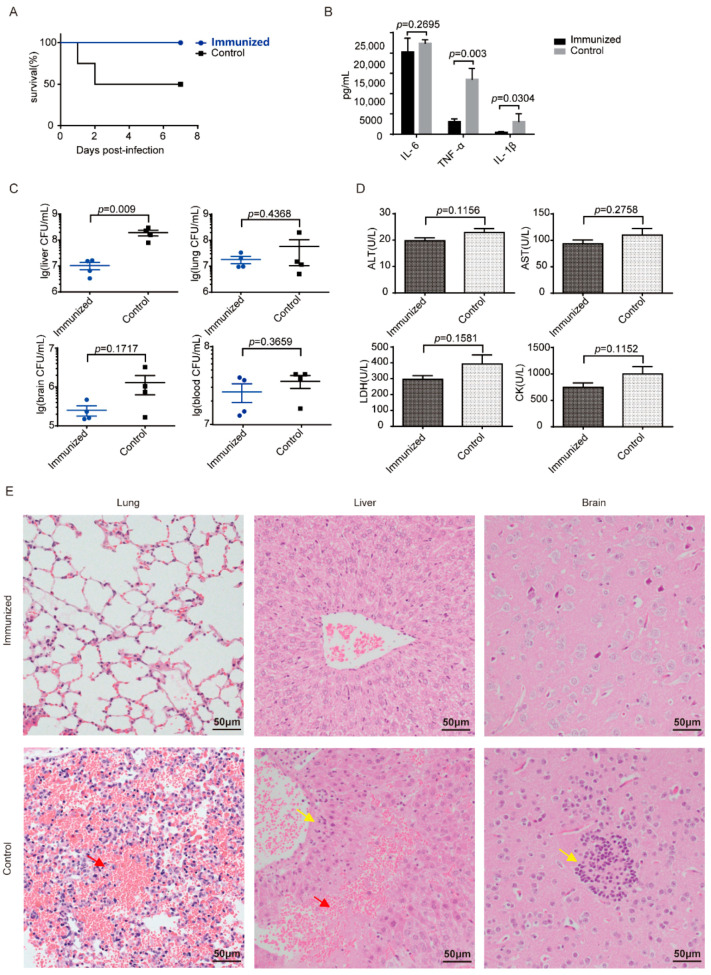
Homologous protection conferred by css-SC19. (**A**) Survival of mice infected with SC19. A total of 14 days following the booster immunization, ten mice from each group were intraperitoneally infected with SC19 (4 × 10^8^ CFU) and monitored daily for 14 days post-infection. Another 5 mice in each group were infected with SC19 (4 × 10^8^ CFU), and 6 h after infection, the blood was collected via cardiac puncture for bacterial load, tissue damage index, and cytokines detection. The lung, liver, and brain were also collected for bacteria load detection and H&E staining. (**B**) For the inflammatory cytokine evaluation, the blood was prepared into plasma and then using U-Plex^TM^ Chemiluminescent ELISA (MSD, USA). (**C**) The bacteria burden in the blood, lungs, liver, and brain. (**D**) The blood chemistry was detected using a VITALAB SE Chemistry Analyzer. (**E**) H&E staining of tissues from mice at 6 h post-infection. Congestion in the lung and liver is indicated by a “red arrow”, and cell degeneration and necrosis are indicated by a “yellow arrow”. Each experimental group was replicated three times.

To verify whether css-SC19 could confer protection against SC19 infection, challenge experiments were conducted. A total of 100% of the immunized mice survived SC19 infection and exhibited no obvious clinical symptoms, whereas all mice in the placebo group succumbed within 48 h and exhibited clinical signs, such as slow responses to stimuli, ruffled hair coat, and tearful eyes (Figure 4A). Additionally, the bacteria burden of SC19 in various tissues was notably reduced, particularly in the liver (Figure 4C). Moreover, the corresponding tissue damage index was alleviated (Figure 4D), and the histopathologic observation revealed minimal lesions in immunized mice after SC19 infection. At the same time, the placebo group showed extensive diffuse hemorrhagic spots in the lung, liver, and brain, as well as degeneration or even necrotic foci of the liver and brain cells and infiltration of inflammatory cells in the lung (Figure 4E). The placebo group showed an obvious inflammatory cytokine storm, but the levels of inflammatory cytokines of IL-1β and TNF-α were relatively lower in the immunized group (Figure 4B). These results indicated that the immunization of css-SC19 provided a comprehensive defense ability for mice against SC19.

### 3.5. Δcps/ssna-msly (P353L)-SC19 Confers Protection against Type 9 Serotypes S. suis and Prevents Meningitis

**Figure 5:**
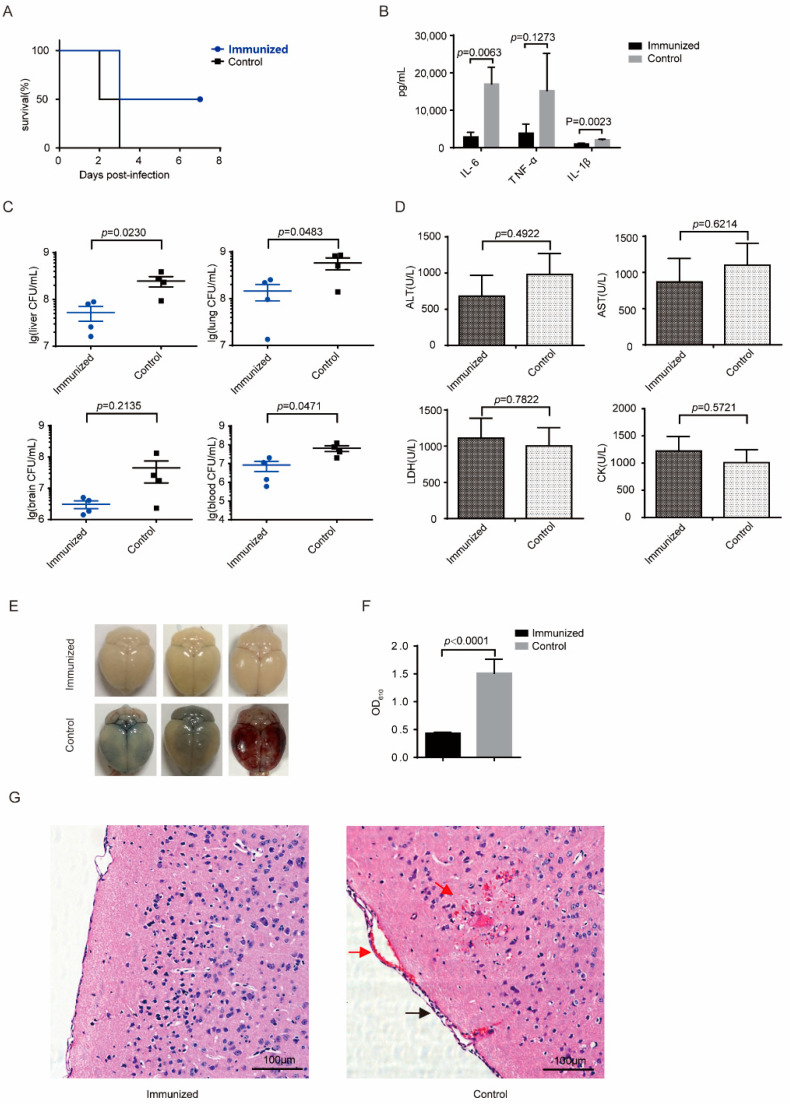
Heterologous protection conferred by css-SC19. Two weeks post-booster immunization, ten mice per group were intraperitoneally infected with SS9 strain S29 (5 × 10^6^ CFU) and were monitored daily for 14 days post-infection. Another five mice in each group were infected with S29 (5 × 10^6^ CFU); 24 h after infection, blood was collected via cardiac puncture for bacterial load, tissue damage index, and inflammatory cytokine detection. The lung, liver, and brain were collected for bacteria load detection and H&E staining. (**A**) Survival of mice infected with SS9 strain S29 (5 × 10^6^ CFU). (**B**) For the inflammatory cytokine evaluation, the blood was prepared into plasma and then using U-Plex^TM^ Chemiluminescent ELISA (MSD, USA). (**C**) Bacteria burden in the blood, lungs, liver, and brain. (**D**) Blood chemistry was detected using a VITALAB SE Chemistry Analyzer. (**E**–**G**) Evaluation of blood–brain barrier integrity of mice infected with meningitis strain S29 (5 × 10^6^ CFU) by Evans blue assay and H&E staining. (**E**) Representative images of brains stained by Evans blue. (**F**) The absorbance spectroscopy at 610 nm of Evans blue extracted from stained brains. (**G**) The H&E staining was performed on brains from mice at 60 h post-infection of S29. Congestion in the brain was indicated by a “red arrow”, while infiltration of inflammatory cells was indicated with a “black arrow”. Each experimental group was replicated three times.

To evaluate the potential of css-SC19 to provide effective protection against heterologous *S. suis* infection, S29 challenge experiments were performed. In the placebo group, mice began to perish within 48 h, displaying purulent secretion in the canthus and neurological symptoms such as circling and head tilting, and all succumbed within 72 h, resulting in a 100% mortality rate. In contrast, some immunized mice exhibited mild canthus secretion at 48 h and perished at 72 h, but 50% survived without displaying any clinical signs (Figure 5A). Meanwhile, the bacteria load retrieved from the blood, lungs, and liver was significantly lower than that of the placebo group (Figure 5B). Although there were no discernible differences in blood biochemical indexes between immunized and placebo-treated mice (Figure 5D), slight pathological changes were evident upon histopathological observation of the placebo-treated mice (Supplementary Figure S1). Additionally, substantially decreased levels of inflammatory factors were also observed (Figure 5B). These findings indicated that css-SC19 could induce cross-protection against S29.

In addition, given that the S29 strain can cause meningitis in mice with typical neurological symptoms, we utilized Evans blue to quantify the blood–brain barrier damage. The mice of the placebo group exhibited visible penetration of Evans blue at 60 h post-infection, whereas the brains of the immunized mice remained unstained (Figure 5E). The quantity analysis of Evans blue extracted from the brain confirmed that the BBB damage in placebo-treated mice was significantly more pronounced than in immunized mice (Figure 5F). In parallel, the histopathologic observation revealed focal hemorrhage, meningeal thickening, inflammatory cell infiltration, and hemorrhage appeared in the brains of the control mice, while no obvious lesions were observed in the brains of the immunized mice (Figure 5G). These results suggested that css-SC19 can mitigate the blood–brain barrier damage caused by S29 infection and prevent the onset of meningitis.

### 3.6. Distinguish the Sera of S. suis-Infected Mice from Immunized Mice

**Figure 6:**
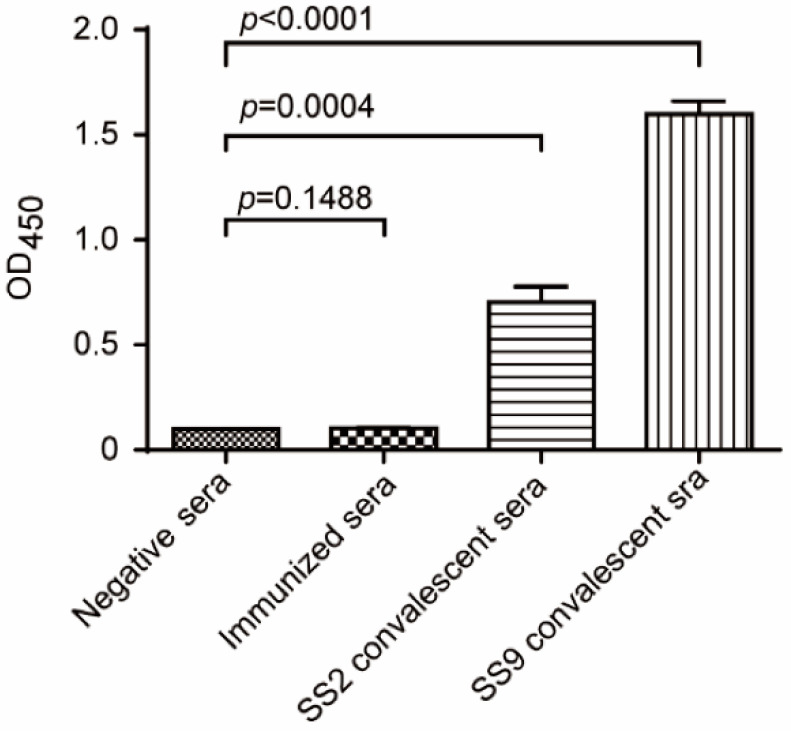
Differentiation of immunized mice or *S. suis*-infected mice by SsnA antibody detection. Two weeks after immunization of css-SC19 or infection of non-lethal doses of different genotypes of *S. suis*, the mouse sera was collected and determined by SsnA-specific ELISA.

To investigate whether the immunized mice could be differentiated from *S. suis*-infected mice, the SsnA antibody levels of immunized mice, SS2-infected mice, SS9-infected mice, and placebo-treated mice were measured by ELISA. Both SS2 and SS9 convalescent sera exhibited a significant reaction to SsnA, whereas sera from the css-SC19-immunized mice showed comparable levels to negative sera (Figure 6). The result indicated that the immunization of css-SC19 can be distinguished from the wild-type *S. suis* infection, thus facilitating immune status assessment and pathogen decontamination.

## 4. Discussion

There are many serotypes of *S. suis*, among which SS2 is the most prevalent and virulent, followed by SS9, which has brought great losses to the pig industry in Europe and is becoming increasingly prevalent in China [29]. Due to the lack of cross-protection among different serotypes of *S. suis*, it is a challenge to develop effective vaccines. Currently, the main strategy involves screening proteins with strong immunogenicity and broad protective properties across all *S. suis* serotypes. In recent years, a great number of candidate antigen proteins for a *S. suis* subunit vaccine have been identified, among which the immunoglobulin M-degrading enzyme (Ide_Ssuis_) [30] and peptidyl isomerase (PrsA) [31] have been reported to provide cross-protection against SS2 and SS9 infections. Another appealing strategy is the glycoconjugate vaccine, composed of CPS linked to an immunogenic carrier protein. While glycoconjugate vaccines were successfully used in combating encapsulated human pathogens [32], the development of glycoconjugate vaccines capable of resisting different *S. suis* serotypes is still under investigation [33]. In addition, attenuated vaccines, which can proliferate in vivo and elicit a robust immune response, have shown promise. For instance, an attenuated mutant, 2015033, was reported to provide cross-protection against different sequence types in CC1 [34]. XS045, a naturally attenuated type-5 strain isolated by Jiang X et al., represents the first reported attenuated vaccine candidate capable of cross-protecting against SS2 and SS9 infections [35], offering promising prospects for the development of *S. suis* vaccines.

Gene deletion offers a safer approach for developing attenuated vaccines. The critical step in constructing these vaccines involves selecting and knocking out key virulence genes. In this study, the key virulence gene *cps* of the SC-19 strain is knocked out to reduce the virulence and expose the potential universal antigens encapsulated by capsules, which is a risky strategy because, although knocking out *cps* will significantly reduce the virulence of the SC-19 strain, many studies believe that CPS is essential for vaccine-mediated immunoprotection [36]. Nevertheless, it is worth trying to achieve protection against different serotypes of *S. suis*. Moreover, a point substitution P353L in the *sly* gene results in SLY losing its hemolytic activity while retaining immunogenicity. In addition, the virulence gene *ssna*, which is associated with immune evasion in *S. suis*, was also knocked out. Given that SsnA exists widely in many serotypes of *S. suis* [37], it can serve as a marker to distinguish vaccine strains from wild-type strains. In the follow-up, the pre-immunization status can be evaluated by detecting SsnA-specific antibodies, enabling a more effective utilization of vaccines.

Safety is usually the most concerning issue of attenuated vaccines. The cytotoxicity experiment of A549 cells in this study proved that the css-SC19 strain had no obvious toxicity in vitro. For the in vivo test, the injection of 4 × 10^9^ CFU of css-SC19 did not cause any disease or death in mice, whereas the experimental dose for assessing animal protection ranges from 10^7^ to 10^9^ CFU per animal. These results proved that the genetically engineered vaccine strain exhibited high safety and could serve as a potential candidate for a live-attenuated *S. suis* vaccine.

On this basis, we further analyzed the immune efficacy of css-SC19 as an attenuated vaccine. After immunization, significant humoral immunity and cellular immunity were induced, as well as opsonizing antibodies, which have been found critical for protective immunity against infection. Moreover, the immunized mice exhibited suppressed inflammatory reactions, reduced tissue damage, and 100% survival following SC19 infection. Simultaneously, it mitigated the inflammatory responses and tissue damage caused by the SS9 strain infection, especially reducing the incidence of blood–brain barrier damage and meningitis, and finally protected 50% of mice from death. It indicated that the deletion of *cps* might expose the universal protective antigen capable of cross-protecting against SS9 infection. Whether the candidate strain of attenuated vaccine provides cross-protection against other serotypes or different STs is worth further exploring. The lower protection of SS9 compared to SC19 may be attributed to the stronger pathogenicity of the SS9 strain in mice, as evidenced by the 2-log lower LD_50_ of SS9 compared to SC-19. To elevate the protection of SS9, we will attempt to express SS9 protective antigens in the genome of css-SC19. Additionally, experiments in pigs were not conducted in this study. Since the protection observed in mice does not fully reflect the protection in pigs, it is essential to confirm the in vivo protection of css-SC19 in the natural host.

In summary, our results demonstrated that immunization with css-SC19, an attenuated mutant with a capsular gene deletion, not only inhibited the lethal STSLS but also conferred immunity to both SS2 and SS9 in mice, which indicates that css-SC19 has great potential for development as a universal attenuated vaccine against *S. suis*.

## Data Availability

All data are available in the article or from the corresponding author upon reasonable request.

## Supplementary Materials

The following supporting information can be downloaded at: https://www.mdpi.com/article/10.3390/vaccines12030283/s1, Table S1: Primers used for the construction of Δ*cps/ssna*-m*sly* (P353L)-SC19. Figure S1: H&E staining performed on tissue samples from mice infected with SS9 strain at 24 h post-infection.

## References

[1] Gottschalk M., Segura M., Xu J. (2007). Streptococcus suis infections in humans: The Chinese experience and the situation in North America. Anim. Health Res. Rev..

[2] Goyette-Desjardins G., Auger J.P., Xu J., Segura M., Gottschalk M. (2014). Streptococcus suis, an important pig pathogen and emerging zoonotic agent-an update on the worldwide distribution based on serotyping and sequence typing. Emerg. Microbes Infect..

[3] Huong V.T., Ha N., Huy N.T., Horby P., Nghia H.D., Thiem V.D., Zhu X., Hoa N.T., Hien T.T., Zamora J. (2014). Epidemiology, clinical manifestations, and outcomes of Streptococcus suis infection in humans. Emerg. Infect. Dis..

[4] Wertheim H.F., Nghia H.D., Taylor W., Schultsz C. (2009). Streptococcus suis: An emerging human pathogen. Clin. Infect. Dis..

[5] Fittipaldi N., Segura M., Grenier D., Gottschalk M. (2012). Virulence factors involved in the pathogenesis of the infection caused by the swine pathogen and zoonotic agent Streptococcus suis. Future Microbiol..

[6] Hatrongjit R., Fittipaldi N., Gottschalk M., Kerdsin A. (2020). Tools for Molecular Epidemiology of Streptococcus suis. Pathogens.

[7] Hill J.E., Gottschalk M., Brousseau R., Harel J., Hemmingsen S.M., Goh S.H. (2005). Biochemical analysis, cpn60 and 16S rDNA sequence data indicate that Streptococcus suis serotypes 32 and 34, isolated from pigs, are Streptococcus orisratti. Vet. Microbiol..

[8] Lun Z.R., Wang Q.P., Chen X.G., Li A.X., Zhu X.Q. (2007). Streptococcus suis: An emerging zoonotic pathogen. Lancet Infect. Dis..

[9] Bi Y., Li J., Yang L., Zhang S., Li Y., Jia X., Sun L., Yin Y., Qin C., Wang B. (2014). Assessment of the pathogenesis of Streptococcus suis type 2 infection in piglets for understanding streptococcal toxic shock-like syndrome, meningitis, and sequelae. Vet. Microbiol..

[10] Wisselink H.J., Veldman K.T., Van den Eede C., Salmon S.A., Mevius D.J. (2006). Quantitative susceptibility of Streptococcus suis strains isolated from diseased pigs in seven European countries to antimicrobial agents licensed in veterinary medicine. Vet. Microbiol..

[11] Segura M., Fittipaldi N., Calzas C., Gottschalk M. (2017). Critical Streptococcus suis Virulence Factors: Are They All Really Critical?. Trends Microbiol..

[12] Segura M. (2015). Streptococcus suis vaccines: Candidate antigens and progress. Expert. Rev. Vaccines.

[13] Roy D., Auger J.P., Segura M., Fittipaldi N., Takamatsu D., Okura M., Gottschalk M. (2015). Role of the capsular polysaccharide as a virulence factor for Streptococcus suis serotype 14. Can. J. Vet. Res..

[14] Smith H.E., Damman M., van der Velde J., Wagenaar F., Wisselink H.J., Stockhofe-Zurwieden N., Smits M.A. (1999). Identification and characterization of the cps locus of Streptococcus suis serotype 2: The capsule protects against phagocytosis and is an important virulence factor. Infect. Immun..

[15] Lecours M.P., Gottschalk M., Houde M., Lemire P., Fittipaldi N., Segura M. (2011). Critical role for Streptococcus suis cell wall modifications and suilysin in resistance to complement-dependent killing by dendritic cells. J. Infect. Dis..

[16] Takeuchi D., Akeda Y., Nakayama T., Kerdsin A., Sano Y., Kanda T., Hamada S., Dejsirilert S., Oishi K. (2014). The contribution of suilysin to the pathogenesis of Streptococcus suis meningitis. J. Infect. Dis..

[17] Jacobs A.A., Loeffen P.L., van den Berg A.J., Storm P.K. (1994). Identification, purification, and characterization of a thiol-activated hemolysin (suilysin) of Streptococcus suis. Infect. Immun..

[18] Lin L., Xu L., Lv W., Han L., Xiang Y., Fu L., Jin M., Zhou R., Chen H., Zhang A. (2019). An NLRP3 inflammasome-triggered cytokine storm contributes to Streptococcal toxic shock-like syndrome (STSLS). PLoS Pathog..

[19] de Buhr N., Neumann A., Jerjomiceva N., von Kockritz-Blickwede M., Baums C.G. (2014). Streptococcus suis DNase SsnA contributes to degradation of neutrophil extracellular traps (NETs) and evasion of NET-mediated antimicrobial activity. Microbiology.

[20] Li M., Cai R.J., Li C.L., Song S., Li Y., Jiang Z.Y., Yang D.X. (2017). Deletion of ssnA Attenuates the Pathogenicity of Streptococcus suis and Confers Protection against Serovar 2 Strain Challenge. PLoS ONE.

[21] Zhao J., Pan S., Lin L., Fu L., Yang C., Xu Z., Wei Y., Jin M., Zhang A. (2015). Streptococcus suis serotype 2 strains can induce the formation of neutrophil extracellular traps and evade trapping. FEMS Microbiol. Lett..

[22] Zhang A., Chen B., Yuan Z., Li R., Liu C., Zhou H., Chen H., Jin M. (2012). HP0197 contributes to CPS synthesis and the virulence of Streptococcus suis via CcpA. PLoS ONE.

[23] Wang X.N., Wang L., Zheng D.Z., Chen S., Shi W., Qiao X.Y., Jiang Y.P., Tang L.J., Xu Y.G., Li Y.J. (2018). Oral immunization with a Lactobacillus casei-based anti-porcine epidemic diarrhoea virus (PEDV) vaccine expressing microfold cell-targeting peptide Co1 fused with the COE antigen of PEDV. J. Appl. Microbiol..

[24] Luo Y., Dorf M.E. (2001). Isolation of mouse neutrophils. Curr. Protoc. Immunol..

[25] Zhang M., Mao Y., Ramirez S.H., Tuma R.F., Chabrashvili T. (2010). Angiotensin II induced cerebral microvascular inflammation and increased blood-brain barrier permeability via oxidative stress. Neuroscience.

[26] Wisselink H.J., Vecht U., Stockhofe-Zurwieden N., Smith H.E. (2001). Protection of pigs against challenge with virulent Streptococcus suis serotype 2 strains by a muramidase-released protein and extracellular factor vaccine. Vet. Rec..

[27] Baums C.G., Bruggemann C., Kock C., Beineke A., Waldmann K.H., Valentin-Weigand P. (2010). Immunogenicity of an autogenous Streptococcus suis bacterin in preparturient sows and their piglets in relation to protection after weaning. Clin. Vaccine Immunol..

[28] Buttner N., Beineke A., de Buhr N., Lilienthal S., Merkel J., Waldmann K.H., Valentin-Weigand P., Baums C.G. (2012). Streptococcus suis serotype 9 bacterin immunogenicity and protective efficacy. Vet. Immunol. Immunopathol..

[29] Dekker N., Bouma A., Daemen I., Vernooij H., van Leengoed L., Wagenaar J.A., Stegeman A. (2017). Effect of Simultaneous Exposure of Pigs to Streptococcus suis Serotypes 2 and 9 on Their Colonization and Transmission, and on Mortality. Pathogens.

[30] Seele J., Hillermann L.M., Beineke A., Seitz M., von Pawel-Rammingen U., Valentin-Weigand P., Baums C.G. (2015). The immunoglobulin M-degrading enzyme of Streptococcus suis, IdeSsuis, is a highly protective antigen against serotype 2. Vaccine.

[31] Jiang X., Yang Y., Zhou J., Liu H., Liao X., Luo J., Li X., Fang W. (2019). Peptidyl isomerase PrsA is surface-associated on Streptococcus suis and offers cross-protection against serotype 9 strain. FEMS Microbiol. Lett..

[32] Bottomley M.J., Serruto D., Safadi M.A., Klugman K.P. (2012). Future challenges in the elimination of bacterial meningitis. Vaccine.

[33] Goyette-Desjardins G., Calzas C., Shiao T.C., Neubauer A., Kempker J., Roy R., Gottschalk M., Segura M. (2016). Protection against Streptococcus suis Serotype 2 Infection Using a Capsular Polysaccharide Glycoconjugate Vaccine. Infect. Immun..

[34] Li Z., Chang P., Xu J., Tan C., Wang X., Bei W., Li J. (2019). A Streptococcus suis Live Vaccine Suppresses Streptococcal Toxic Shock-Like Syndrome and Provides Sequence Type-Independent Protection. J. Infect. Dis..

[35] Jiang X., Yang Y., Zhu L., Gu Y., Shen H., Shan Y., Li X., Wu J., Fang W. (2016). Live Streptococcus suis type 5 strain XS045 provides cross-protection against infection by strains of types 2 and 9. Vaccine.

[36] Wisselink H.J., Stockhofe-Zurwieden N., Hilgers L.A., Smith H.E. (2002). Assessment of protective efficacy of live and killed vaccines based on a non-encapsulated mutant of Streptococcus suis serotype 2. Vet. Microbiol..

[37] Fontaine M.C., Perez-Casal J., Willson P.J. (2004). Investigation of a novel DNase of Streptococcus suis serotype 2. Infect. Immun..

