# Supplementary material for: A Streptococcus suis Strain Δcps/ssna-msly (P353L)-SC19 Provides Cross-Protection against Serotypes 2 and 9 Strain Challenges in a Mouse Model
Source: Vaccines (Basel). 2024 Mar 8;12(3):283. doi: 10.3390/vaccines12030283 (PMC10974582; doi:10.3390/vaccines12030283)
Supplement: Supplementary file 1 [file vaccines-12-00283-s001.zip › vaccines-2749622-supplementary.pdf]

Table S1. Primers used for the construction of Δ*cps*/*ssna*-m*sly* (P353L)-SC19.

Primer	Sequence	Function	Target sequence
ECORI-E-1	AAAGAATTCGGCTCGTGCTATATTCTCTTG G	Amplication of left arm sequence of *cpsEF* gene for construction of the isogenic mutant with thermosensitive suiside vector pSET4S	481 bp
RONGHE-EF-2	GAATCTTTTTCATAACAGCTCCTCCACTATT TC		
RONGHE-EF-1	GAAATAGTGGAGGAGCTGTTATGAAAAAG ATTC	Amplication of right arm sequence of *cpsEF* gene for construction of the isogenic mutant with thermosensitive suiside vector pSET4S	495 bp
BAMHI-F-2	AAAGAATCCGGTTTGCCACAGCCTGTG		
DSsnA-LF1	GGCGAATTCTGTTCGCATCAGTCGTAG	Amplication of left arm sequence of *ssna* gene for construction of the isogenic mutant with thermosensitive suiside vector pSET4S	650 bp
DSsnA-LR1	GAACCAGGGCGTATCTCTTCATATAAAACT CCTTTTTGTTAT		
Dssna-RL1	GAAAGATCGCCGTGTAATCATTTTTTGAGC TTGAAAGCATGAC	Amplication of right arm sequence of *ssna* gene for construction of the isogenic mutant with thermosensitive suiside vector pSET4S	658 bp
Dssna-RR1	CGAGGATCCCAATTTCAACCTCGGTCGCTC		
SSNA-jd-1	GTTTTAAGTTGTTGAGCTGAAACAGTC	Primers for PCR identification of *ssna* mutant and sequencing	3303 bp (mutant) 4587 bp (WT)
SSNA-jd-2	GTCGCCATAAATGGCGGATAG		
CPSEF-F	GTTTATCCTGAAATACGATTGTGC	Primers for PCR identification of *cpsEF* mutant and sequencing	1891 bp (mutant) 3689 bp (WT)
CPSEF-R	TAATACACTTTATTATCTACCCCATTG		
SLY-ATG-F	ATGAGAAAAAGTTCGCACTTGATTT	Primers for *sly* amplication and sequencing	1491 bp
SLY-wuTAA-R	CTCTATCACCTCATCCGCATACTGT		

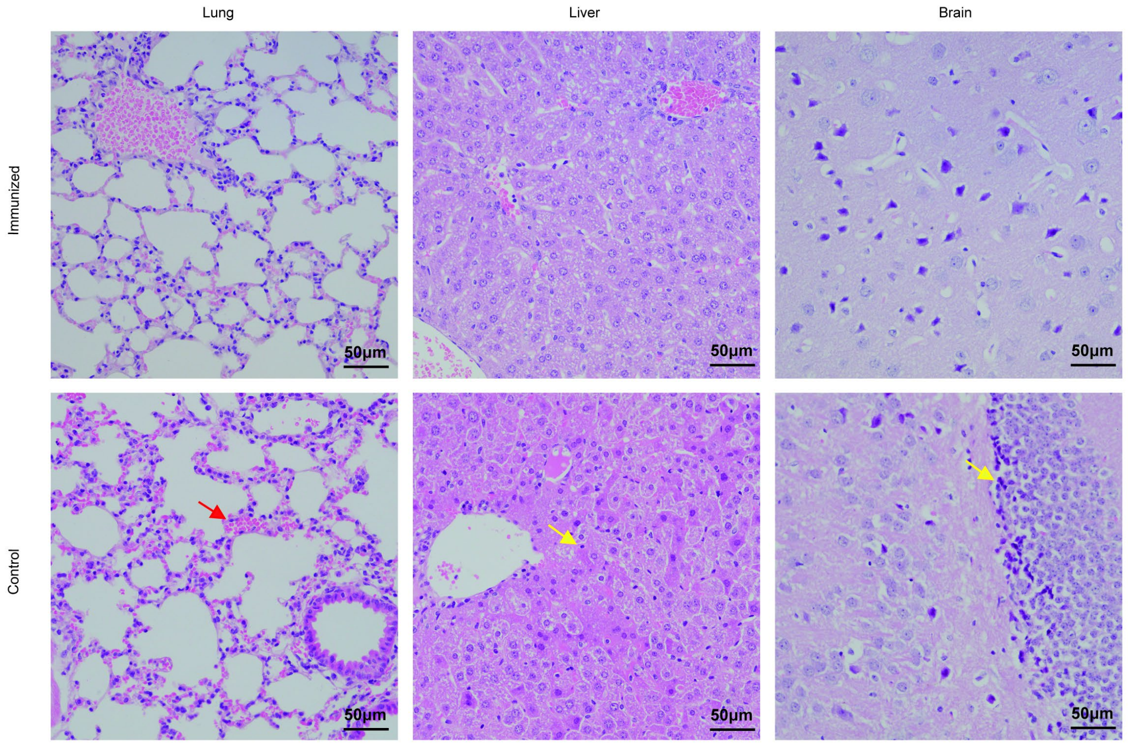

Supplementary Figure 1.H&E staining performed on tissue samples from mice infected with SS9 strain at 24 h post-infection. Congestion in the lung and liver was indicated by a “red arrow”, while cell degeneration and necrosis were indicated by a “yellow arrow”.

A

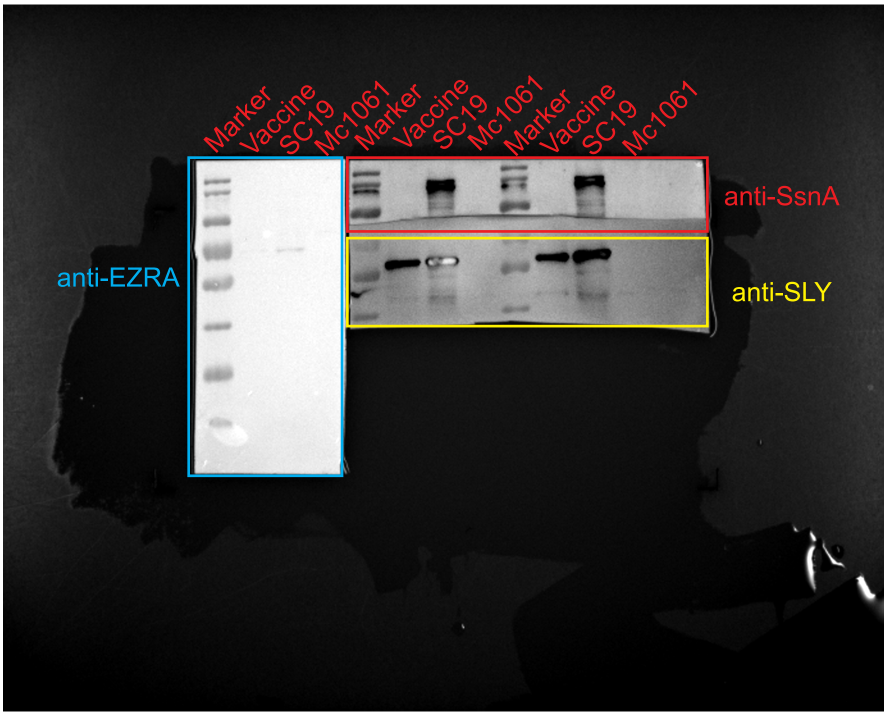

B

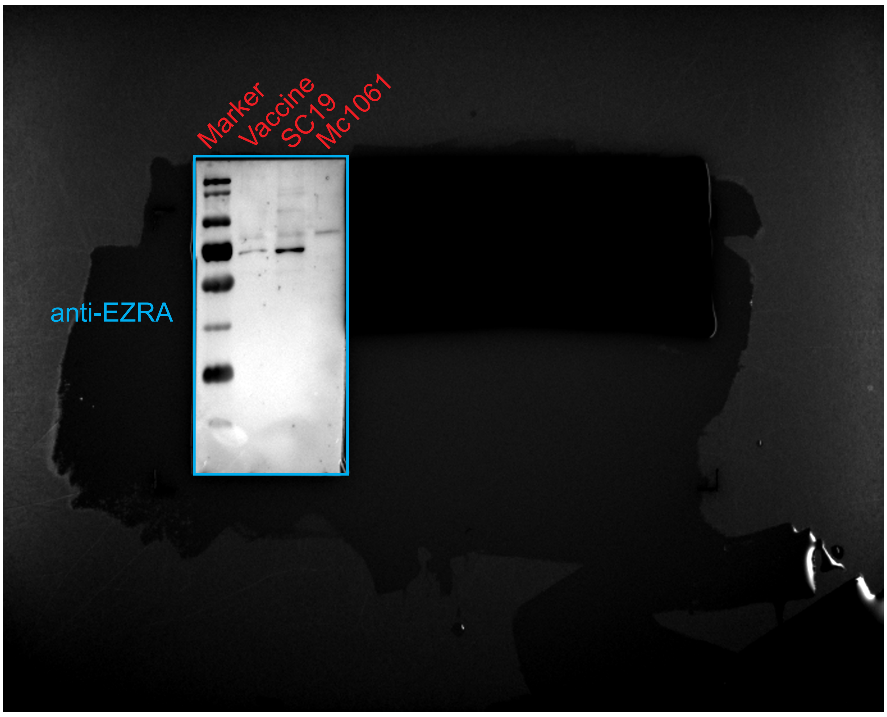

Fig1C. original blot figure

(A):The expression of SsnA and SLY was detected by western blot, while EZRA served as a loading control, Mc1061 served as a negative control. The exposure time is 10 seconds.(B):The expression of EZRA.The exposure time is 20 seconds.
